# From current to future projections: modeling habitat suitability changes for *Hibiscus syriacus* L. in China using MaxEnt under climate change

**DOI:** 10.3389/fpls.2025.1551684

**Published:** 2025-06-02

**Authors:** Jing Xu, Yanghui Zhao, Chuncheng Wang, Yadan Yan, Yafeng Wen

**Affiliations:** ^1^ College of Landscape Architecture, Central South University of Forestry and Technology, Changsha, Hunan, China; ^2^ Hunan Big Data Engineering Technology Research Center of Natural Protected Areas Landscape Resources, Changsha, Hunan, China; ^3^ Yuelushan Laboratory Carbon Sinks Forests Variety Innovation Center, Changsha, Hunan, China

**Keywords:** climate change, global warming, MaxEnt, suitable habitat, *Hibiscus syriacus* L.

## Abstract

*Hibiscus syriacus* L. (Malvaceae) is widely cultivated for its ornamental value and diverse applications in food, medicine, and textiles. Despite its extensive use, the key environmental factors and geographic patterns influencing its habitat suitability remain poorly understood. We applied the MaxEnt model to assess the current and projected future distribution of *H. syriacus* using 185 occurrence records and 20 environmental variables. Results showed that the current suitable habitat area covered 188.81 × 10^4^ km². Temperature and precipitation played a crucial role in shaping the present geographical distribution of *H. syriacus* populations. Projections indicated that by the 2050s, the total suitable habitat area would expand, with the SSP585 scenario demonstrating the most substantial increase. However, a general decline was expected by the 2070s. The potential distribution, primarily concentrated in Hunan Province, was projected to shift southwestward. Migration patterns and habitat changes were primarily driven by substantial variations in temperature and precipitation. These findings highlight the impact of climate change on the habitat suitability of *H. syriacus* and offer a scientific basis for determining planting zones and strategies.

## 1 Introduction

According to the Intergovernmental Panel on Climate Change Sixth Assessment Report (Lei et al., 2022), the impacts and risks of global warming are becoming increasingly complex, leading to a range of irreversible effects on ecosystems and human societies (Kikstra et al., 2022). In particular, climate change has a pronounced influence on plants, primarily through shifts in temperature and precipitation patterns. These changes disrupt the availability of energy and water necessary for plant growth, thereby impairing physiological and ecological processes and, in severe cases, causing reduced growth or plant mortality. Such disruptions inevitably threaten global food security and the production of economically significant crops (Jaskani and Khan, 2021; Piao et al., 2019; Zhao et al., 2021). Over the past 30 years, researchers have quantified and elucidated the reduction in crop yields and the contraction of suitable habitats caused by climate disasters by analyzing disaster indices (Hu et al., 2024), statistical data (Shi et al., 2021), information diffusion technology (Rezaei et al., 2023), and production function models (Kang et al., 2009). Overall, the impact of climate change on agricultural production is mainly evident in fluctuations in crop yields caused by extreme weather events and temperature changes, with global warming and changes in precipitation patterns exacerbating this impact.

*Hibiscus syriacus* L. (Malvaceae), native to China, is a multifunctional shrub valued for its ornamental beauty, medicinal applications, and role in agriculture and food production. The diverse uses of *H. syriacus* make it a crop of significant economic importance. The production and distribution of *H. syriacus* rely on the nursery industry, which is traditionally sensitive to environmental changes. Previous studies on *H. syriacus* have primarily focused on its medicinal value (Yang et al., 2020) and chemical extracts (Yang et al., 2019), with limited attention being paid to the environmental factors restricting its distribution and its adaptability to climate change. This gap in understanding has led to mixed results when introducing *H. syriacus* to various regions (Jing, 2015; Liu et al., 2021; Feng et al., 2022), thereby revealing a lack of knowledge about its specific climatic requirements. The insufficient research on the environmental conditions necessary for its growth has contributed to poor outcomes in many regions, including stunted development, low survival rates, irregular flowering, and increased vulnerability to frost damage, in many regions (Feng et al., 2021; Wang, 2018). These challenges are largely attributed to mismatches between the plant and local climate conditions, which remain inadequately studied. Nurseries and horticultural enterprises are particularly vulnerable to these uncertainties (Whittet et al., 2016). Without clear guidance on optimal climate conditions, site selection errors often lead to wasted resources and production delays (Fernandez et al., 2022). Although key factors such as temperature, precipitation, and seasonal fluctuations significantly influence the growth of *H. syriacus*, their specific impacts remain poorly understood. These knowledge gaps have hindered the introduction of *H. syriacus* to new regions, thereby limiting its broader adoption and commercial potential.

In species distribution modeling, various algorithms, including generalized linear models, random forests, and MaxEnt, are employed to predict and understand the spatial patterns of species distribution (Ahmadi et al., 2023; Shabani et al., 2016). Each method offers distinct advantages and suits to specific data types and availability. Among these, MaxEnt has gained widespread application because of its flexibility and robustness in analyzing presence-only data (Wang et al., 2024; Merow et al., 2013; Wen et al., 2024). The model has demonstrated reliable predictive performance, particularly in scenarios involving fragmented or incomplete data. Its capability to integrate presence-only data with environmental variables makes it a valuable tool for projecting potential habitat shifts under changing climate conditions (Phillips and Dudík, 2008; Yang et al., 2020; Duan et al., 2022). These applications are essential for understanding species’ ecological adaptability and improving agricultural and horticultural management practices. By analyzing relationships between species distribution and environmental factors, MaxEnt effectively identifies ecological thresholds and adaptation ranges. This capability supports site selection, habitat suitability analysis, and strategic planning. As a result, MaxEnt serves as a valuable tool to address challenges related to species introduction and sustainable cultivation in variable climatic contexts (Elith et al., 2011).

In this work, we utilized the MaxEnt model to assess the impact of climate change on the potential habitat suitability of *H. syriacus*. The primary research objectives are as follows: (1) to quantify the key environmental drivers shaping the distribution of *H. syriacus* and define its ecological suitability thresholds; (2) to simulate habitat range changes, including expansion or degradation patterns and centroid migration trends, under future climate scenarios; and (3) to identify the priority planting habitat for *H. syriacus* based on current and future suitability distributions, and propose planting strategies that address the challenges posed by climate change.

## 2 Materials and methods

### 2.1 Species occurrence data

**Figure 1:**
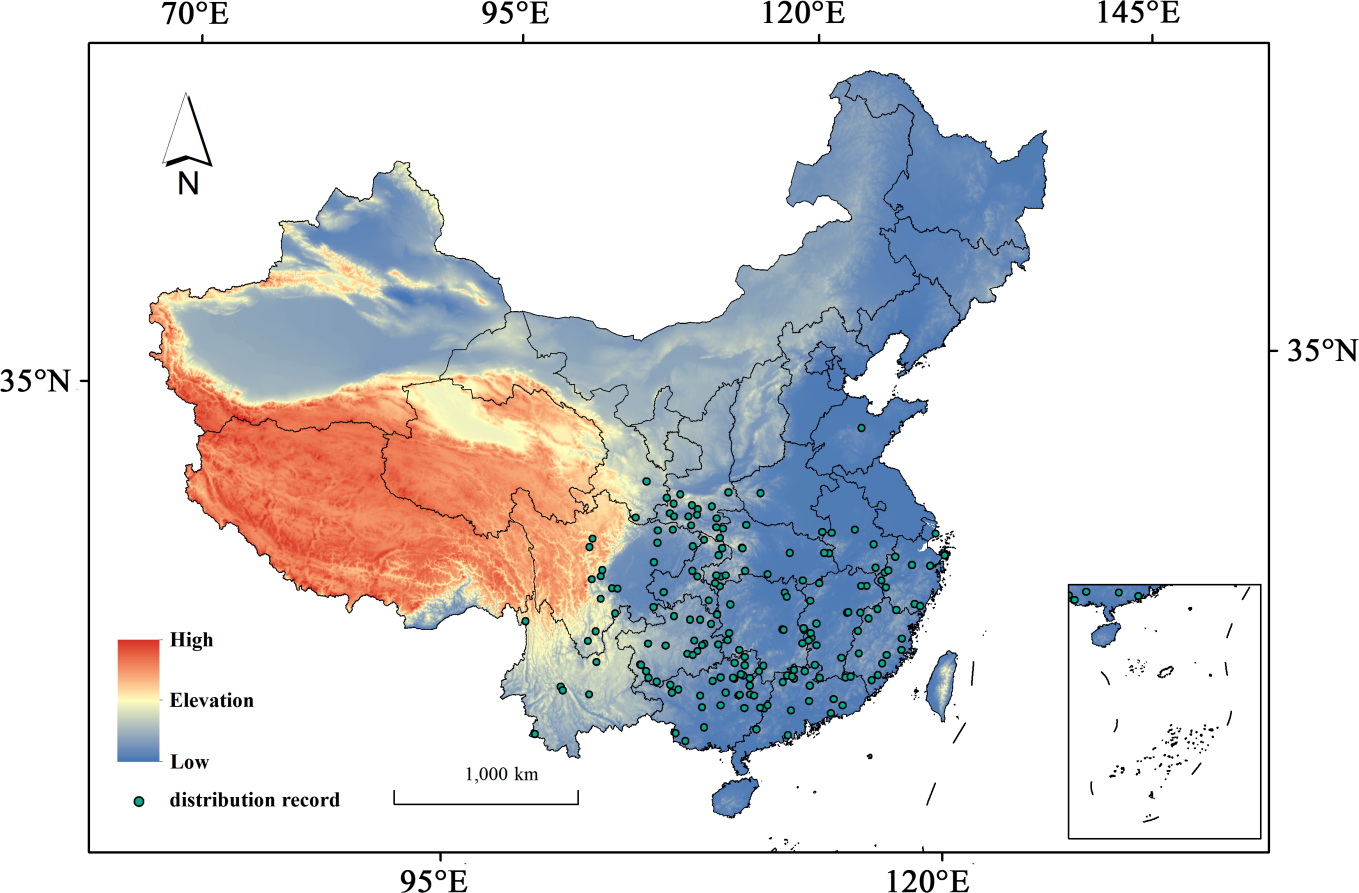
Distribution records of *H. syriacus* in China.

This study used “*Hibiscus syriacus*” as a keyword to collect and process its distribution data systematically. Specimen records were obtained from the Global Biodiversity Information Facility (GBIF, https://www.gbif.org/); in particular, 1,839 raw occurrence points for *H. syriacus* were obtained by setting the filter “Country or Area” to China. We conducted overlay analyses to cross-reference land-use classifications and remove redundant or inaccurate records associated with water bodies, buildings, and roads by integrating species distribution data from the Chinese Virtual Herbarium (CVH, http://www.cvh.ac.cn/), Google Maps (Google Maps, http://www.gditu.net/), and 2020 land-use data (DCRES, https://www.resdc.cn/) into ArcMap 10.8.1; in this way, overfitting in species distribution models caused by clustering effects can be avoided. This step aimed to identify and retain naturally occurring distribution points while excluding potential cultivated locations. The occurrence records were refined using SDMToolbox v2.4 to ensure spatial independence of the input data and achieve optimal model performance, thereby ensuring that each 2.5-arc-minute grid cell (approximately 4.64 km at the equator) contained only one occurrence record. Ultimately, 185 valid occurrence points were obtained for subsequent analyses (**Figure 1**).

### 2.2 Environmental data

Current environmental data (1970—2000) and future climate data for two periods (2050s and 2070s), with a resolution of 2.5 minutes, were downloaded from the Global Climate Database (WorldClim, http://www.worldclim.org/). These data includes 19 climate variables related to precipitation and temperature (BIO1–BIO19) and one elevation variable (ELV). For future climate data, we utilized simulations based on the BCC-CSM2-MR model data, covering three radiative forcing scenarios: low (SSPs126), medium (SSPs245), and high (SSPs585). The BCC-CSM2-MR model is recommended for conducting short-term climate forecasting and for examining climate change trends within China (Wu et al., 2021). These scenarios reflect different socio-economic trajectories and their impacts on climate policy and radiative forcing levels.

**Figure 2:**
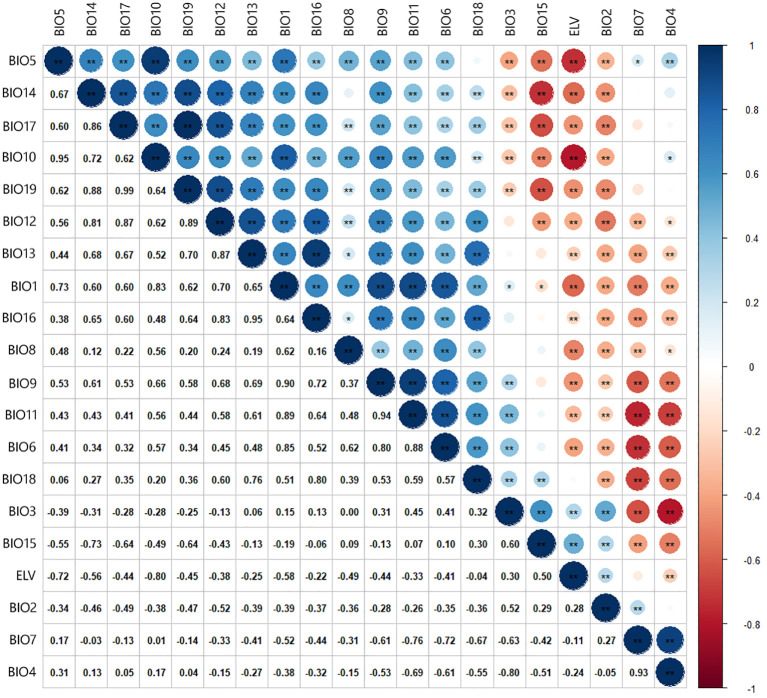
Correlation of environment variables.

Preliminary simulations were conducted to analyze the contribution rates of environmental factors to minimize model overfitting effectively. Pearson correlation analysis was utilized (**Figure 2**) (Hosseini et al., 2024; Khajoei Nasab et al., 2020). Factors with a correlation coefficient greater than 0.75 were excluded to reduce redundancy and improve model accuracy (Dormann et al., 2013; Zhang et al., 2022). The selected environmental factors for the final dataset include BIO2, BIO4, BIO6, BIO8, BIO12, BIO14, and elevation (ELV) (**Table 1**).

**Table 1:** Percentage contributions and permutation importance of the variables included in the MaxEnt models for *H. syriacus*.

Code	Environmental Variables	Unit	Percent Contribution	Permutation Importance
BIO2	Mean Diurnal Range	°C × 10	3.1	5.1
BIO4	Temperature Seasonality	× 100	8.9	41.4
BIO6	Min Temperature of Coldest Month	°C × 10	22.4	14.4
BIO8	Mean Temperature of Wettest Quarter	°C × 10	3.8	12.6
BIO12	Annual Precipitation	mm	34.6	9.7
BIO14	Precipitation of Driest Month	mm	23.6	5
ELV	elevation	m	3.5	11.8

### 2.3 Model simulation

We utilized the MaxEnt model version 3.4.4 to simulate the potential distribution of *H. syriacus* based on species records and environmental factors. Previous studies have shown that using default settings can result in suboptimal models (Fourcade et al., 2014; Morales et al., 2017; Lissovsky and Dudov, 2021); therefore, we explored various combinations of regularization multipliers and proportions of test sets. Our analysis revealed that using 70% of the distribution points for training and the remaining 30% for testing, along with a regularization multiplier of 0.5, provided the best model performance, thereby ensuring high predictive accuracy and generalization capability. The default “autofeatures” including linear, hinge, quadratic, threshold, and product features, were retained. The relative importance of environmental variables was analyzed using the Jackknife test. Model performance was evaluated using the receiver operating characteristic (ROC) curve, and the area under this curve (AUC) was calculated to quantify model accuracy (Merow et al., 2013). AUC values range from 0 to 1, with values higher than 0.8 indicating excellent model accuracy (Morasca and Lavazza, 2020).

A threshold-based method was used to classifying habitat suitability (Morales et al., 2017). Suitable areas were classified into four categories: non-suitable (0–0.2), low suitability (0.2–0.4), moderate suitability (0.4–0.6), and high suitability (0.6–1) (Bora and Saikia, 2024; Barboza et al., 2024). Potential suitable areas for the contemporary period, 2050s, and 2070s were projected onto a map of China, and the area of potential suitable regions for each period was calculated using a field calculator.

### 2.4 Spatial pattern changes and centroid movement in suitable areas

A threshold of 0.5 for species presence probability was established to delineate potential suitable areas for *H. syriacus*. Then the model outputs were reclassified using ArcMap10.8.1, thereby enabling the visualization of shifts in the spatial patterns of habitat suitability for *H. syriacus*. We utilized SDMtoolbox v2.6 to compare and calculate the geographical centroid of species under different climate scenarios. We also evaluated the binary habitat suitability for species to categorize the areas into three classifications: expansion, unchanged, and loss (Fois et al., 2018).

## 3 Results

### 3.1 Evaluation of model simulation results of *H. syriacus*

**Figure 3:**
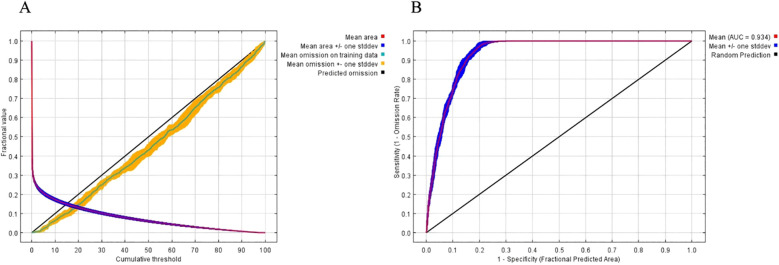
Model accuracy evaluation. AICc values of parameter combinations were calculated using ENMeval. AICc (Akaike information criterion correction) reflects model optimization, with DAICc = 0 indicating the optimal parameter combination. Feature categories include L (linear), Q (quadratic), H (hinge), P (product), T (threshold), and combinations such as LQ, LQH, LQHP, and LQHPT. **(A)** The ROC verification curve of Maxent model; **(B)** Jackknife test of the importance of variables.

The ROC curve is a widely used tool for evaluating the balance between sensitivity and specificity in predictive models. In this study, the ROC curve approaches the top left corner, indicating high diagnostic accuracy and a low false positive rate (**Figure 3**). The AUC value for the training set is 0.934, which reflects the robustness and reliability of the model in predicting the potential distribution of *H. syriacus*. These results highlight the effectiveness of using the MaxEnt model to assess species distributions under current and future climate scenarios.

### 3.2 Critical environmental factors influencing the distribution of *H. syriacus*

The contribution rates of seven environmental factors to the prediction of contemporary habitat suitability for *H. syriacus* using the MaxEnt model are summarized in **Table 1**. Annual precipitation (BIO12) emerges as the most significant factor, accounting for 34.6% of the total contribution. Ranked second and third are precipitation of the driest month (BIO14) and the minimum temperature of the coldest month (BIO6), contributing 14.4% and 13.9%, respectively. Other factors, including the standard deviation of temperature seasonality (BIO4), mean temperature of the wettest quarter (BIO8), mean diurnal range (BIO2), and elevation (ELV), contribute 8.9%, 3.8%, 3.5%, and 3.1%, respectively. The combination of precipitation-related factors (BIO12 and BIO14) contributes 58.2% to the model, whereas temperature-related factors account for 38.3%. These results highlight the dominant role of precipitation in determining the habitat suitability of *H. syriacus*, followed by the influence of temperature-related factors.

**Figure 4:**
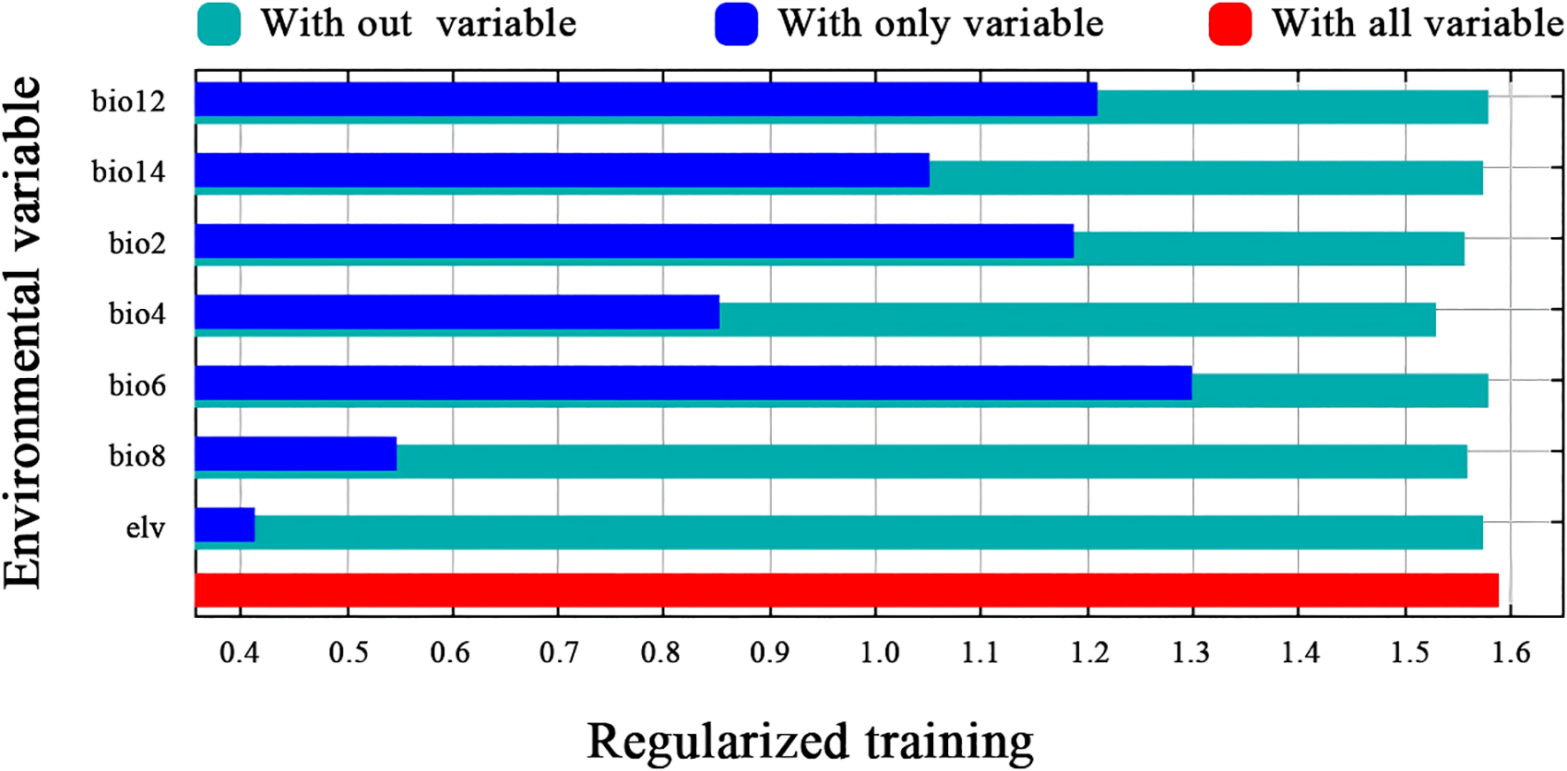
Jackknife analysis of environmental variables.

The results of the jackknife test (**Figure 4**) indicated that BIO6, BIO12, and BIO14 are the most influential factors affecting the predictive accuracy of the model. Each factor was evaluated in isolation to assess its corresponding contribution. BIO6 exhibits a high training gain in the univariate model, thereby underscoring its pivotal role in predicting the geographical distribution of *H. syriacus*. Similarly, BIO12 and BIO14 show significant training gains, emphasizing the importance of precipitation variability in simulating plant distribution. Moreover, the exclusion of BIO4 led to a marked reduction in predictive performance. These findings indicate that BIO6, BIO12, BIO14, and BIO4 are the key environmental factors influencing the distribution of *H. syriacus*. Moreover, this analysis provides insights into the primary environmental drivers shaping the species’ distribution under current climatic conditions.

**Figure 5:**
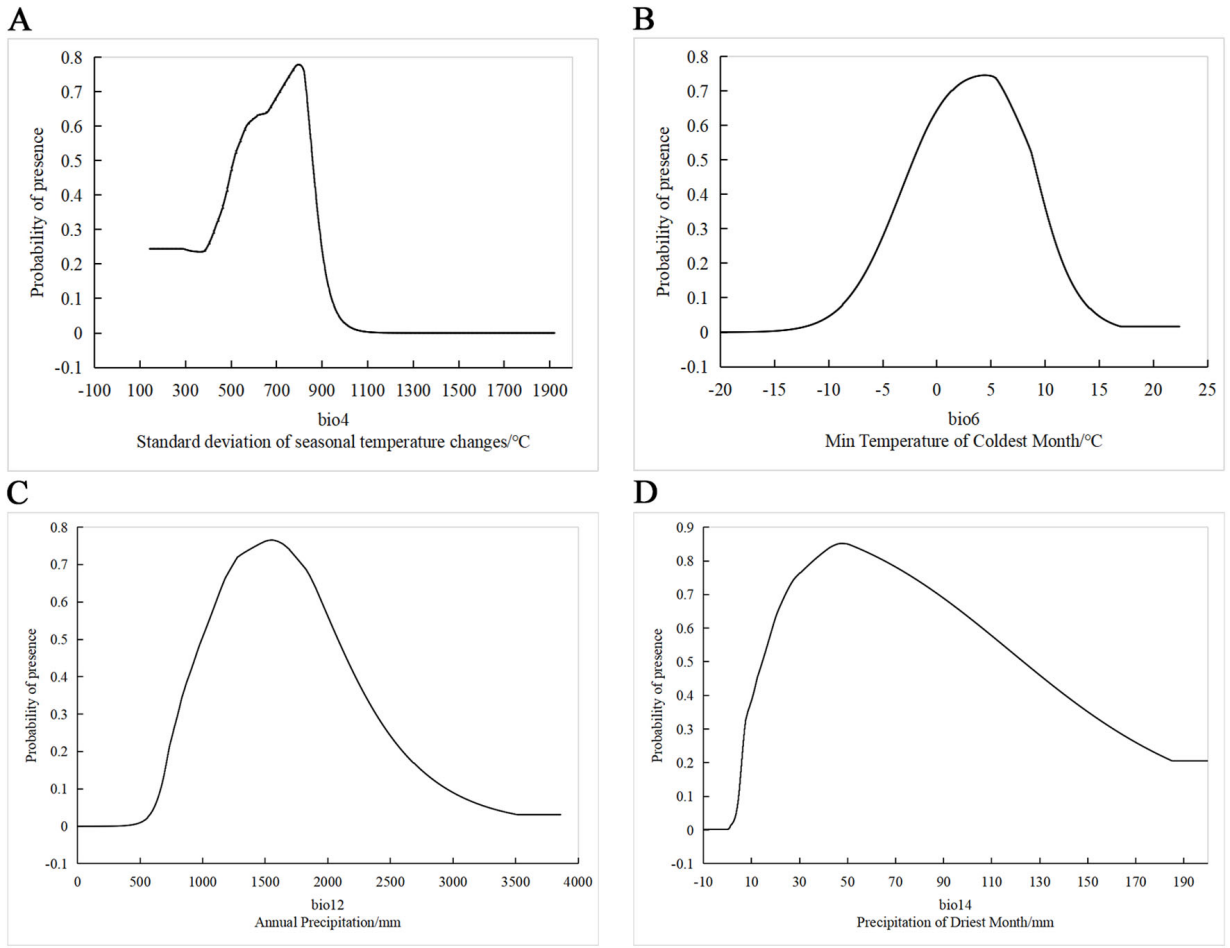
Response curves of main bioclimatic factors. The x-axis represents the range of environmental variable values, and the y-axis represents the predicted probability of species presence. **(A)** The relationship between bio4 (Standard deviation of seasonal temperature changes/°C) and species presence probability. **(B)** The effect of BIO6 (Minimum Temperature of Coldest Month/°C) on species presence probability. **(C)**The relationship between BIO12 (Annual Precipitation/mm) and species presence probability. **(D)** The impact of BIO14 (Precipitation of Driest Month/mm) on species presence probability.

The response curves of various environmental factors (**Figure 5**) show the ranges that support the presence of *H. syriacus*. The optimal ranges were defined based on a probability threshold of ≥0.6. For BIO12, the suitable annual precipitation range is 1100 mm to 1950 mm, with the recorded highest probability of occurrence being between 1500 and 1600 mm. For BIO14, precipitation during the driest month ranges from 18.7 mm to 104 mm, with the concentration of optimal values being between 40 and 60 mm. BIO6 demonstrates a suitable temperature range for the coldest month between −0.8 and 7.7°C, with the highest probability being around 4°C to 6°C. The response curve for BIO4 reveals a distinct unimodal pattern, with the habitat suitability peaking when the seasonality index is near 800, within an optimal range of 570 to 840.

### 3.3 Predictions of current suitable habitat

**Figure 6:**
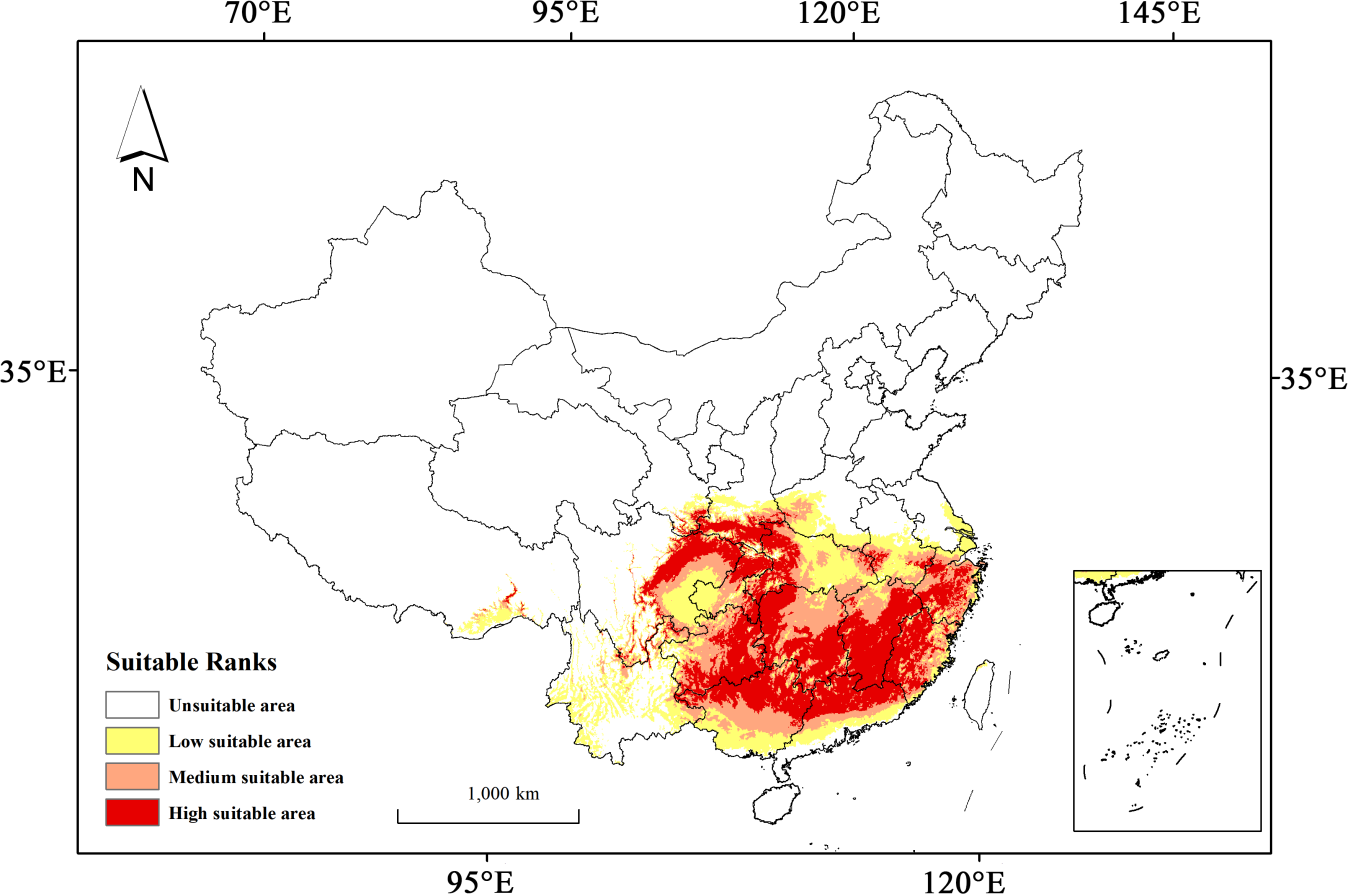
Potential habitat prediction for *H. syriacus* under current climatic scenario.

Under the present climate scenario, the total potential habitat of *H. syriacus* is estimated to extend across approximately 188.81×10^4^ km^2^ (**Table 2**), accounting for 19.67% of the total land area in China. This habitat is divided into low, medium, and high suitability zones, with areas of 58.85×10^4^ km^2^, 57.94×10^4^ km^2^, and 72.02×10^4^ km^2^, respectively. The high suitability zones account for 31.17% of the overall suitable habitat. In terms of geography, the regions suitable for *H. syriacus* primarily include Hubei, Hunan, and the southwestern part of Henan in central China; Jiangxi, Fujian, Zhejiang, and southern Anhui in eastern China; Guangdong and Guangxi in southern China; the eastern parts of Sichuan; Guizhou, Chongqing, and Yunnan in southwestern China; and the southern part of Shaanxi in northwestern China (**Figure 6**). The distribution of *H. syriacus* specimens aligns closely with the predicted potential areas of the model because most specimens are located in medium and high suitability zones. This consistency underscores the predictive accuracy and reliability of the model.

**Table 2:** Prediction area of potential suitable distribution areas for *H. syriacus* in different periods.

Suitability Category	Current	2050			2070		
		SSPs126	SSPs245	SSPs585	SSPsp126	SSPs245	SSPs585
Unsuitable area	773.62	773.66	774.3	767.96	773.74	775.88	778.29
Low suitable area	58.85	58.11	52.8	55.01	47.85	57.35	43.61
Medium suitable area	57.94	62. 27	46.03	57. 31	64.06	55.96	57.38
High suitable area	72.02	68.38	89.3	82.14	76.78	73.24	83.14
Total suitable area	188.81	188.76	188.13	194.46	188.69	186.55	184.13
Compared to current		-0.05	-0.68	5.65	-0.12	-2.26	-4.68

Unit (104km2).

### 3.4 Future alterations of appropriate habitat area

Under future climate scenarios for the 2050s and 2070s (**Table 2**), the total suitable area for *H. syriacus* is projected to exhibit a general declining trend, except under the SSP585 scenario in the 2050s, where an increase of 5.65×10^4^ km^2^ can be observed. In the 2050s, changes in suitable areas under the SSP126 and SSP245 scenarios are minimal, whereas a notable expansion is projected under the SSP585 scenario. By contrast, the 2070s are projected to show a reduction in suitable area across all scenarios, with the largest decrease of −4.68×10^4^ km^2^ occurring under SSP585.

**Figure 7:**
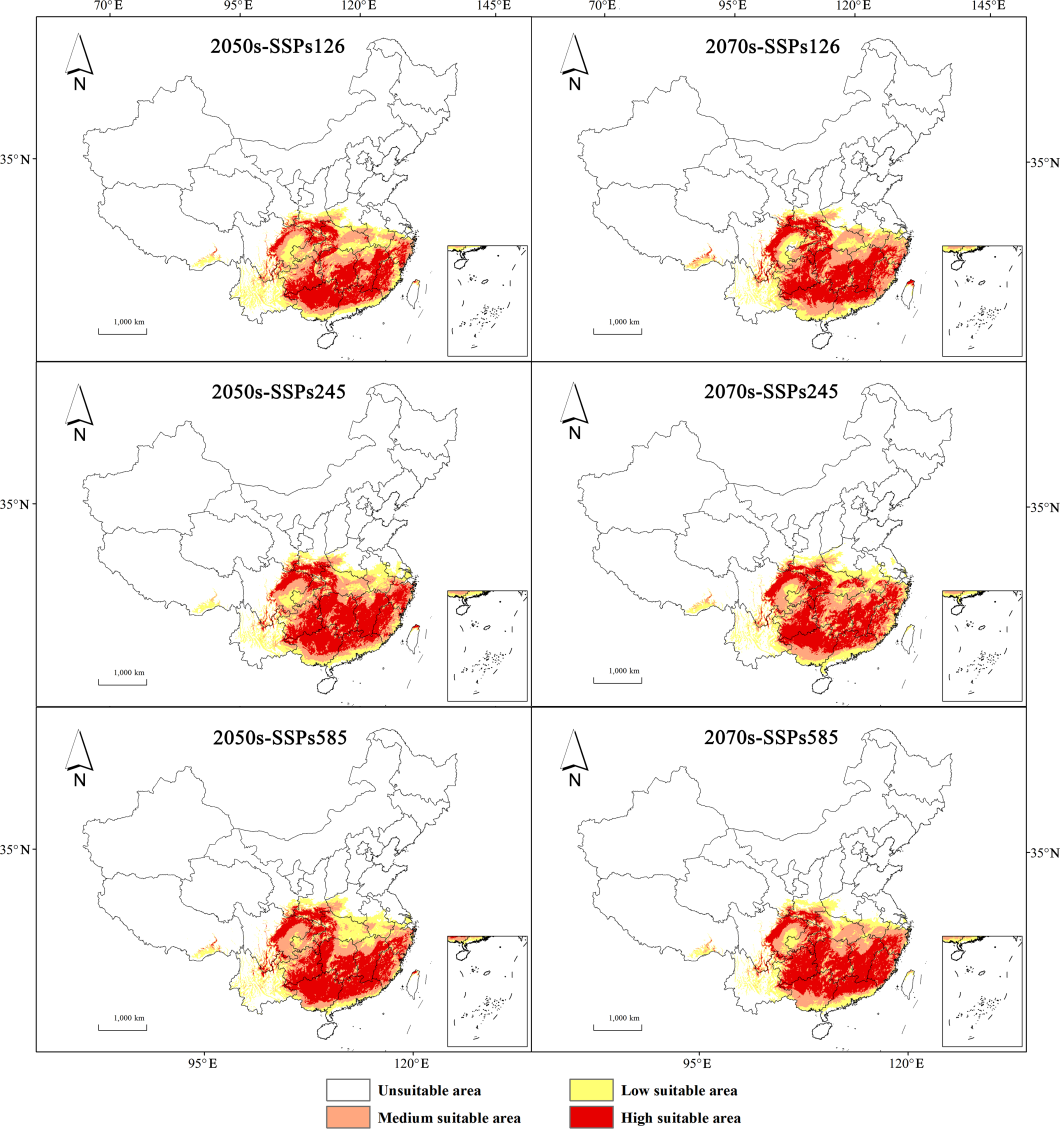
Potential habitat prediction for *H. syriacus* under future climatic scenario.

Compared with contemporary levels, the area of low suitability for *H. syriacus* is expected to decline, whereas the area of high suitability is projected to demonstrate an increasing trend. Comparisons between projections for the 2050s and 2070s reveal greater fluctuations in high and low suitability areas under the SSP585 scenario. In terms of geography, medium and high suitability areas are primarily projected to be located in Hunan, Jiangxi, Fujian, Guangdong, Guangxi, and Guizhou, whereas low suitability areas are expected to expand mainly to the west and north (**Figure 7**).

### 3.5 Spatial patterns of *H. syriacus* distribution under different future scenarios

**Figure 8:**
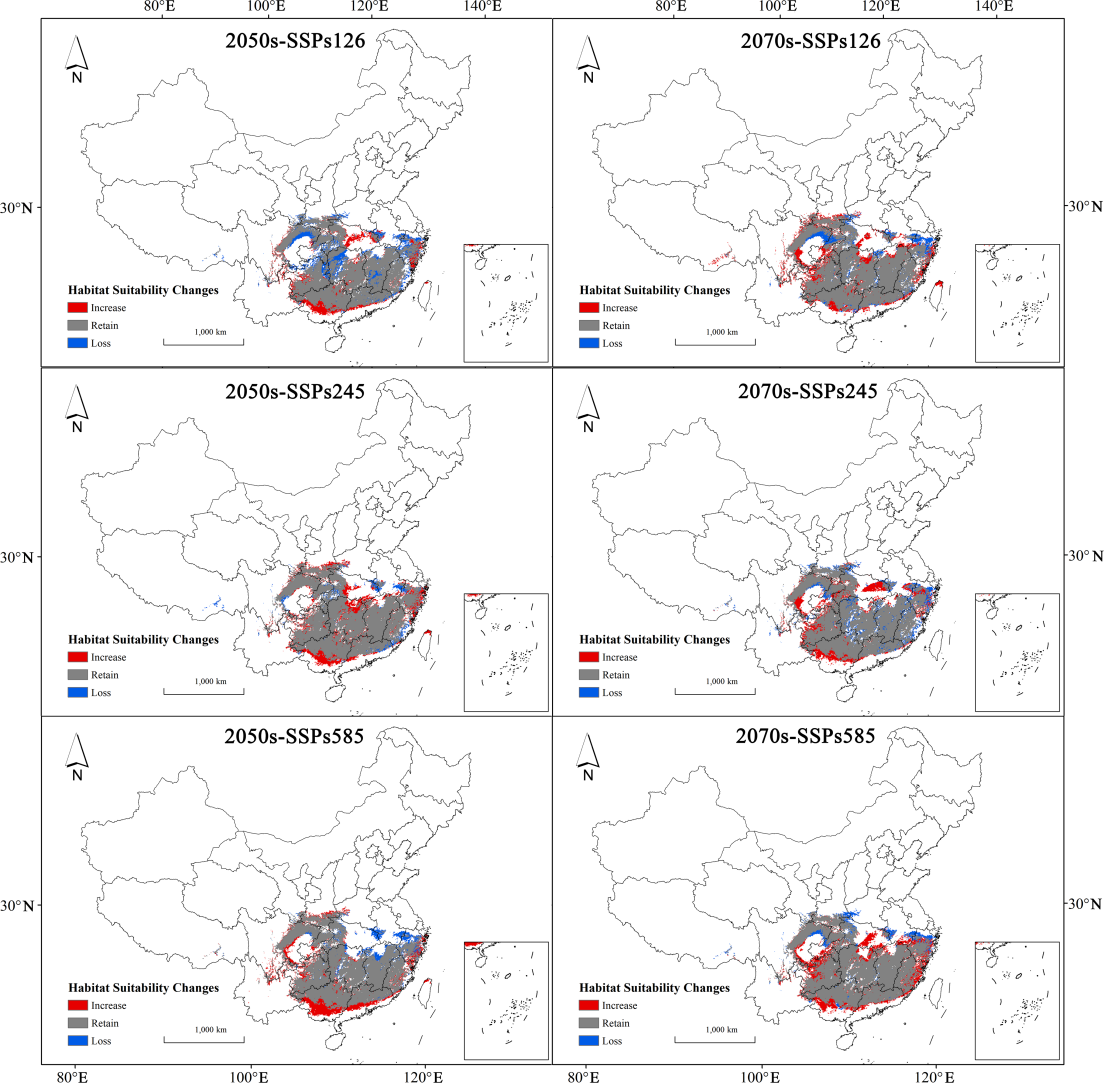
*H. syriacus* habitat suitability expansion and contraction under future climate scenarios.

Based on the spatial changes in suitable areas for *H. syriacus* under various future climate scenarios (**Table 3**), projections for the 2050s and 2070s indicate high retention rates of suitable habitats, ranging from 86.33% to 95.26%, predominantly in regions such as Hunan, Jiangxi, Guizhou, Guangxi, Guangdong, Zhejiang, Fujian, and Chongqing (**Figure 8**). Under SSP126 and SSP245, the retention rates exceed 90%, indicating strong resilience to moderate climate changes. However, under SSP585, retention rates decline slightly, with loss rates ranging from 6.69% to 13.68%. Habitat expansion was observed across all scenarios, particularly in the 2070s, with expansion areas ranging from 11.96 × 10^4^ km^2^ to 19.15 × 10^4^ km^2^, corresponding to increase rates of 11.79% to 18.87%. These expansions occurred primarily along the edges of current suitable regions, particularly in southern and eastern China, including parts of Jiangxi, Hunan, and Zhejiang. SSP585 projected the largest expansion, suggesting a potential northward and eastward habitat shift under extreme conditions. Despite these expansions, significant habitat losses were predicted, particularly in the southernmost parts of the range. Losses are concentrated in southern Guangxi, Guangdong, southeastern Fujian, and parts of Hainan, with SSP245 and SSP585 scenarios showing the most pronounced declines. Loss rates vary between 4.74% and 13.68%, depending on the scenario and period.

**Table 3:** Spatial variation of suitable growth areas for *H. syriacus* at different future climate scenarios.

Future climate scenarios	Area (10^4^km^2^)			Change (%)		
	Increase	Retain	Loss	Increase Rate	Retention Rate	Loss Rate
2050s-SSPs126	11.96	87.61	13.88	11.79	86.33	13.68
2050s-SSPs245	17.23	96.67	4.81	16.98	95.26	4.74
2050s-SSPs585	16.53	94.07	7.41	16.29	92.7	7.3
2070s-SSPs126	17.94	92.79	8.7	17.68	91.44	8.57
2070s-SSPs245	15.39	92.09	9.39	15.17	90.75	9.25
2070s-SSPs585	19.15	94.7	6.79	18.87	93.32	6.69

The Increase, Retention, and Loss Rates are derived by dividing the respective areas—increased, retained, and lost—by the total current suitable area and are expressed as percentages to indicate the proportional change from the baseline under future climate scenarios.

In summary, although *H. syriacus* demonstrates high resilience under moderate climate scenarios, extreme scenarios, such as SSP585, indicate substantial spatial redistribution. Retained habitats remain concentrated in central and southern regions, such as Hunan, Guizhou, and Chongqing, but overall patterns suggested localized losses alongside significant northward and eastward expansions.

### 3.6 Centroid changes under different climate scenarios

**Figure 9:**
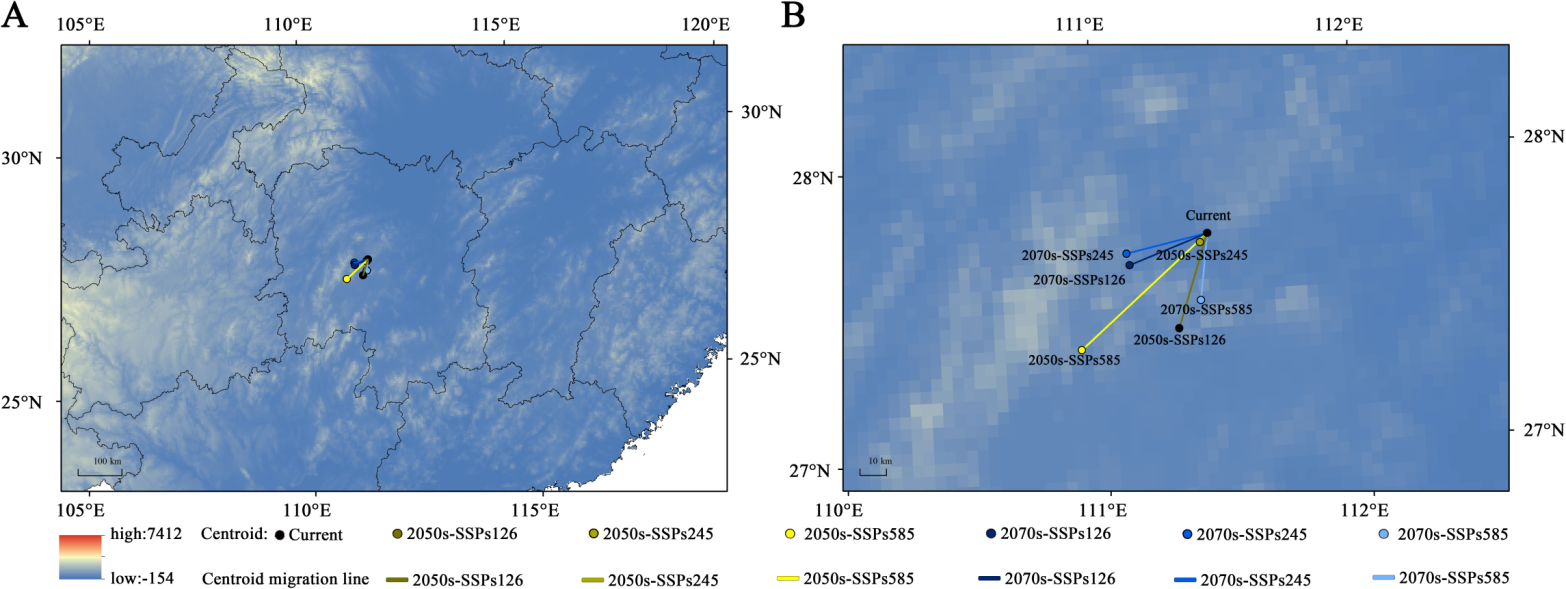
The centroid migration of *H*. *syriacus* under future climate scenario. **(A)** Geographic distribution of the centroid migration of *H*. *syriacus*; **(B)** Zoomed-in view of the centroid migration of *H*. *syriacus*.

The geographical centroid of suitable habitats for *H. syriacus* demonstrated notable migration patterns under different climate scenarios (**Figure 9**). Currently, the centroid is located in Xinhua County, Loudi City, Hunan Province (111°25′12″E, 27°44′25″N). By the 2050s, under the SSP245 scenario, the centroid will migrate slightly within Xinhua County, covering a short distance of 3.27 km (111°22′21″E, 27°42′37″N). By contrast, under the SSP126 and SSP585 scenarios, the centroid would shift by 29.19 and 42.21 km to Shaoyang County (111°17′34″E, 27°25′13″N) and Longhui County, Shaoyang City (110°54′54″E, 27°21′56″N), respectively.

By the 2070s, under the SSP585 scenario, the centroid will move further south, covering 39.64 km to Xinshao County, Shaoyang City (111°22′51″E, 27°30′42″N). In comparison, westward migration can be observed under the SSP126 and SSP245 scenarios, placing the centroid in Xinhua County at (111°6′52″E, 27°38′45″N) and (111°6′17″E, 27°41′09″N), with distances of 20.48 km and 21.93 km, respectively. Across all scenarios, the overall migration distance of the centroid ranges from 3.27 km to 42.21 km, consistently showing a southwestward trend. These findings suggest that future climatic conditions will drive significant shifts in the ecological niche of *H. syriacus*, with the extent of migration depending on the severity of climate scenarios.

## 4 Discussion

### 4.1 Key environmental factors influencing *H. syriacus* distribution

According to the percentage contribution of various environmental factors, precipitation (BIO12 and BIO14) is the most critical factor influencing the distribution of *H. syriacus*, accounting for 58.2% of the total model contribution. In particular, BIO12 contributes 34.6%, This value reflects the humidity conditions of a region throughout the year and directly influences water availability, which is crucial for a species that thrives in moist environments. Based on the response curves of annual precipitation (**Figure 5C**), the suitable range for the growth of *H. syriacus* is between 1000 and 2100 mm, with the optimal conditions being around 1400 mm. According to data from the China Meteorological Administration (https://weather.cma.cn/), regions such as Jiangxi, Zhejiang, Hunan, and Guangdong have annual precipitation ranging from 1100mm to 1600 mm. These regions provide highly suitable environments that closely match the optimal moisture range for *H. syriacus* predicted by the model, further confirming the accuracy and reliability of the model.

Reduced winter precipitation can significantly negatively impact the biomass of perennial plants (Henry et al., 2018). Although *H. syriacus* demonstrates certain drought tolerance, prolonged drought affects its growth and flowering. Therefore, adequate precipitation during the driest month is essential for maintaining normal physiological growth (Winters et al., 2018). The response curve of BIO14 indicates a high probability of presence (P > 0.8) within the 40mm to 60 mm precipitation range (**Figure 5D**). However, the probability begins to decline after 45 mm, ultimately reaching its lowest point of 0.2 at 180 mm. This response to decreasing precipitation likely represents a natural adaptation to the combined effects of environmental conditions. For instance, higher precipitation often correlates with lower temperatures. In the heavy snowfall areas on the Japan Sea side, winter precipitation and temperature have a significant negative correlation, indicating that increased precipitation coincides with decreased temperatures (Long et al., 2021). Consequently, cold winter monsoons bringing low temperatures and high precipitation may have inhibited the growth of *H. syriacus*. Additionally, excessively wet soil in low-temperature conditions increased the risk of root diseases (Yamaguchi et al., 2013). As modeled in this study, the current and future potential distributions of *H. syriacus*, provide crucial insights for its cultivation and management. Given that precipitation significantly impacts its distribution, regions with optimal precipitation patterns, such as parts of Jiangxi, Zhejiang, Hunan, and Guangdong, were identified as prime areas for cultivation. These findings highlight the importance of understanding how current and future precipitation patterns can affect the distribution and growth of *H. syriacus*.

Temperature factors (BIO6, BIO4, and BIO8) contributed a combined value of 35.1% to the distribution model of *H. syriacus*, there by underscoring their critical role in determining its distribution. BIO6 demonstrated the highest presence probability within the 1°C to 7°C range (**Figure 5B**). Temperatures beyond this range significantly reduced the likelihood of *H. syriacus* presence, likely because this range supports efficient metabolic and physiological processes (Aslam et al., 2022). Extreme temperatures, namely, high and low, exert pronounced adverse effects on plant growth (Duncan et al., 2020); for instance, elevated temperatures cause leaf and stem scorching, premature leaf drop, senescence, and inhibited growth (Lutze et al., 2024). These effects extend beyond structural damage to impair photosynthesis and respiration, ultimately shortening the lifecycle of *H. syriacus* and reducing productivity (Bita and Gerats, 2013; Xu et al., 2023). The response curve of BIO4 indicates that *H. syriacus* achieved the highest presence probability when monthly temperature variations were approximately 8% of the annual mean temperature (**Figure 5A**). However, the increase of this index increased to 11% resulted in a sharp decline of the presence probability to zero, This finding highlights *H. syriacus’s* limited adaptability to substantial temperature fluctuations. Moderate temperature variations optimize photosynthetic and energy metabolism processes (Li et al., 2020). Conversely, excessive variations impose physiological stress, thereby destabilizing photosynthetic pigments and reducing the activity of essential enzymes (Yamori et al., 2014). This disruption leads to a decline in photosynthetic efficiency and undermines overall physiological stability (Song et al., 2014; Sharma et al., 2020).

The identification of these optimal climatic ranges addresses the previously observed challenges in introducing and cultivating *H. syriacus*, particularly those caused by mismatched environmental conditions. The survival and growth rates of *H. syriacus* can be significantly improved by establishing precise thresholds, including annual precipitation between 1000 and 2100 mm, precipitation during the driest month ranging from 40 mm to 60 mm, stable temperatures between −2 and 9°C, and seasonal temperature variations within 500 to 900 (**Figure 5**). These findings underscore the necessity of aligning climatic conditions in future introduction efforts to minimize cultivation risks and enhance resource efficiency in targeted regions.

### 4.2 Impact of climate change and adaptation strategies for the potential habitats of *H. syriacus*

*H. syriacus* is a deciduous shrub that thrives in warm and humid environments. Model predictions indicated that the primary suitable areas for *H. syriacus* are located in southeastern China. These regions, situated in the subtropical southeast and parts of the temperate zone, are characterized by warm and humid climates that align well with the ecological preferences of *H. syriacus*. This distribution pattern is consistent with findings from studies on other tropical and subtropical plant species adapted to similar climatic conditions (Zhao et al., 2024; Zhao et al., 2023).

Predictions for the 2050s under SSP126 and SSP245 scenarios suggested minimal changes in suitable habitat area, whereas those for the SSP585 scenario indicate a significant expansion. This finding implies that the warming and increased precipitation associated with high-emission scenarios can benefit the survival of *H. syriacus*. However, all predicted scenarios in the 2070s indicate a decline in suitable habitat areas, with the most pronounced reduction being under SSP585. Low- to medium-suitability areas can be primarily affected by this decline, whereas high-suitability areas continue to expand under SSP585. As global warming accelerates, surface temperatures in China are projected to increases by 2.3°C to 3.3°C by 2050, with winter and spring temperatures increasing more than in other seasons, and average annual precipitation rising by 5% to 7%. The expansion of high-suitability areas may result from rising winter temperatures and increased annual precipitation. This phenomenon can enhance growth conditions for *H. syriacus* without exceeding its optimal thresholds. Nevertheless, this scenario is also expected to lead to more frequent and intense extreme weather events and uneven precipitation distribution (Piao et al., 2010). For instance, the average annual precipitation in the Qinghai–Tibet region was projected to increase by 9.4 mm per decade, whereas that in the southwestern region, it was expected to decrease by 9.6 mm per decade (Ortega-Reig et al., 2017). In parts of Yunnan Province, where the current precipitation during the driest month is only 10–15 mm, further reductions can make these areas unsuitable for *H. syriacus*, thereby resulting in an overall decline in suitable habitat areas.

High-efficiency water-saving irrigation techniques, such as drip irrigation and microsprinkler systems, can be introduced to improve irrigation systems, thereby mitigating the adverse effects of reduced precipitation and rising temperatures on *H. syriacus* cultivation. These technologies significantly reduce water wastage through precise irrigation and prevent issues such as soil drought or waterlogging caused by uneven irrigation, thereby safeguarding the health of *H. syriacus* root systems (Yang et al., 2023). Studies have indicated that drip irrigation can increase water use efficiency by more than 30% in perennial crops in water-scarce regions, while also avoiding root diseases caused by over-irrigation (Kandelous et al., 2012). Additionally, the establishment of rainwater harvesting systems is particularly crucial in drought-prone areas. These systems can store rainfall during wet seasons and alleviate water stress during the driest months, thereby ensuring the growth needs of *H. syriacus*. Comprehensive management strategies are required to address the adverse impacts of rising temperatures. The increasing frequency of extreme heat events may significantly inhibit the growth of *H. syriacus*. High temperatures cause leaf scorching, defoliation, premature senescence, and reduced productivity by impairing photosynthesis and respiration. These effects can be mitigated by employing physical cooling measures, such as the use of shade nets, to provide adequate protection for *H. syriacus* during hot seasons, thereby reducing transpiration rates and minimizing water loss. Additionally, research has shown that mulching techniques can prevent soil moisture evaporation and regulate surface temperatures; in this way, surface temperatures can be kept within the optimal range for *H. syriacus* growth (Ramos et al., 2024). The introduction of smart agricultural technologies, such as sensors and the Internet of Things, is also crucial for addressing changes in temperature and precipitation (Zhang and Guo, 2016). Soil moisture sensors and weather stations can monitor soil and atmospheric conditions in real time, enabling precise irrigation management through smart irrigation systems. This approach prevents physiological stress caused by overirrigation or underirrigation. The widespread application of these technologies, particularly in regions sensitive to climate change, can improve water use efficiency by 20%–40% and mitigate the threats posed by high temperatures and low precipitation to *H. syriacus* growth by dynamically adjusting irrigation schedules (Kader et al., 2019).

In conclusion, under present climatic conditions, regions such as Jiangxi, Zhejiang, Hunan, and Guangdong are characterized by annual precipitation levels ranging from 1100 mm to 1600 mm and moderate temperature fluctuations. Thus, they are rendering suitable for *H. syriacus* cultivation. The suitable zones under the SSP585 scenario, the suitable zones are projected to expand toward southern provinces, including Guangxi and additional areas in Guangdong, because of the increasing precipitation and warming winter temperatures. However, certain regions, including parts of Yunnan, Henan, and Jiangsu, are expected to become barely suitable under future climate conditions. Integrating high-efficiency irrigation techniques, shading and cooling measures, rainwater harvesting systems, and smart agricultural technologies is essential to mitigate these adverse impacts and ensure sustainable cultivation. These strategies address water scarcity and temperature stress, thereby providing practical solutions to safeguard *H. syriacus* plantations under changing climatic conditions.

### 4.3 Spatial change patterns and centroid shift analysis

In this study, the distribution centroid of *H. syriacus* gradually shifted from Xinhua County in Loudi City, Hunan Province, to various counties in Shaoyang City. This migration reflects the geographical response of the species to climate change and highlights its potential ecological adaptation strategies. In particular, centroid migration, which serves a critical biogeographical indicator, is commonly used to assess the sensitivity and adaptive capacity of species to environmental changes (Chandra et al., 2022; Manish, 2022). In this case, the southwestward shift of the centroid is likely driven by regional temperature increases and winter warming. These climatic changes have likely alleviated low-temperature stress while remaining within the optimal ecological thresholds for *H. syriacus*. Additionally, the humid climate of these regions enhances their suitability, further emphasizing the role of warm and humid environments as key factors driving the migration (Neilson et al., 2005). The observed centroid migration provides insights into the niche stability of *H. syriacus*, thereby showcasing its ability to maintain stable distribution patterns and adapt to future climate scenarios.

Under future climate scenarios, ranging from the relatively mild SSP126 to the extreme SSP585, the already warm and humid climates in southwestern China are projected to intensify further (Mokhtar et al., 2020). This climatic intensification increases the ecological suitability of these regions for *H. syriacus*, as indicated by the southwestward shift in the distribution centroid. Despite substantial climatic variations, the persistence of the centroid between Loudi City and Shaoyang City in Hunan Province, demonstrates the ecological niche stability of *H. syriacus* and its capacity to adapt to diverse environmental conditions. These adaptations enable *H. syriacus* to maintain consistent distribution patterns while responding to changes in temperature and precipitation thresholds. The stability observed in this distribution can also be attributed to the buffering effects of the regional climate. High humidity and moderate winter temperatures in southwestern China mitigate the impact of seasonal variability, ensuring favorable conditions for growth and reproduction. This consistency in environmental conditions promotes both improved productivity and resilience to climate-induced stress (You et al., 2021). Consequently, optimizing planting layouts and management strategies in these climatically favorable areas can maximize both ecological and economic outcomes. Such approaches align with the principles of sustainable intensification, thereby ensuring higher yields while minimizing environmental risks (Talaat, 2023).

### 4.4 Research limitation

Several limitations should be acknowledged in our study. First, the results of the MaxEnt model are strongly influenced by the quality and accuracy of the occurrence data. We integrated specimen data, land use information, and Google Maps to verify whether the distribution points represented natural populations, thereby minimizing potential errors. However, some degree of misclassification remains, particularly because of the historical nature of the specimen records and the influence of human activity. Second, our model primarily considers abiotic factors (climate and topography) and does not incorporate biotic interactions (e.g., competition and dispersal limitations) or human impacts (e.g., land-use changes and urbanization). Therefore, the predicted suitable habitats may not fully reflect the actual distribution of the species. Furthermore, while MaxEnt is widely used in species distribution modeling; thus its exclusive use in this study limits the ability to compare results across different modeling approaches and assess model uncertainty comprehensively.

## 5 Conclusion

We predicted the current and future potential distribution of *H. syriacus* under three SSP climate scenarios by using the MaxEnt model. The current suitable habitat is primarily concentrated in central and eastern China. Annual precipitation and precipitation of the driest month were identified as the key environmental drivers shaping its ecological niche. Future projections indicate a southwestward shift and moderate expansion of suitable areas, with the distribution centroid remaining within central Hunan Province. This finding suggests niche stability under climatic change. Priority planting zones were identified in southwestern China, where warm and humid conditions were projected to persist. Conversely, regions such as Henan, Yunnan, and Jiangsu Provinces may experience declining suitability. This projection highlights the need for proactive adjustments in planting layouts and localized environmental management. To address climate-induced challenges, adaptive strategies such as water-saving irrigation, temperature buffering, and precision agriculture are recommended to optimize cultivation and ensure long-term resilience.

## Data Availability

The original contributions presented in the study are included in the article/supplementary material. Further inquiries can be directed to the corresponding author.
